# Brain-gut-microbiota axis: a review on the bidirectional regulatory mechanisms between gut microbiota and brain and their disease interactions

**DOI:** 10.3389/fmicb.2026.1768891

**Published:** 2026-03-27

**Authors:** Hui Shen, Si Yun Wang, Yan Yan Zhao, Jia Lin Zhou, Jie Zhao, Wei Kai Zhu

**Affiliations:** 1 Traditional Chinese Medicine Department, First Affiliated Hospital of Dalian Medical University, Dalian, Liaoning, China; 2 National-Local Joint Engineering Research Center for Drug Research and Development of Neurodegenerative Diseases, Dalian Medical University, Dalian, Liaoning, China; 3 College of Health-Preservation and Wellness, Dalian Medical University, Dalian, Liaoning, China; 4 Institute (College) of Integrative Medicine, Dalian Medical University, Dalian, Liaoning, China

**Keywords:** brain-gut-microbiota axis, gut microbiota, immune activation, microbial metabolites, neural pathways

## Abstract

**Objective:**

To synthesize current evidence on the bidirectional regulatory mechanisms of the Brain-Gut-Microbiota Axis (BGMA), its perturbation by external factors, and its clinical implications for neurodegenerative, psychiatric, metabolic, and gastrointestinal disorders.

**Design:**

Narrative review integrating preclinical and clinical evidence.

**Data sources:**

PubMed/Medline, EMBASE, Cochrane Library searches (2000–2023) using keywords: “brain-gut-axis,” “microbiota,” “dysbiosis,” “neuroinflammation,” “SCFAs,” “neurodegeneration,” “psychobiotics.”

**Results:**

Diet, stress, antibiotics, and environment significantly alter gut microbiota composition (e.g., reducing diversity, shifting Firmicutes/Bacteroidetes (F/B) ratio). Dysbiosis disrupts BGMA communication via: (1) Neural pathways (vagus nerve modulation); (2) Immune activation (cytokine release, neuroinflammation); (3) Microbial metabolites (SCFAs, tryptophan derivatives, TMAO). These disruptions are associated with Alzheimer’s disease (reduced *Faecalibacterium*, amyloid deposition), Parkinson’s (elevated TMAO, *α*-synuclein aggregation), and depression (altered serotonin synthesis), though causality remains to be established in human studies.

**Conclusion:**

The BGMA is a critical mediator of systemic health. Dysbiosis contributes to disease pathogenesis through defined neural, immune, and metabolic pathways. Targeting the microbiota offers novel therapeutic strategies. Future research must prioritize translational studies validating microbial biomarkers and interventions in human cohorts.

## 1 Introduction

The Brain-Gut-Microbiota Axis (BGMA) represents a dynamic, bidirectional communication network integrating the central nervous system (CNS), enteric nervous system (ENS), and the trillions of bacteria, archaea, fungi, and viruses comprising the gut microbiota. Beyond fundamental roles in nutrient metabolism, immune maturation, and gut barrier integrity, the gut microbiota actively modulates neurodevelopment, behavior, and cognition. Dysbiosis—altered microbial composition and function—is implicated in the rising global burden of neurodegenerative [Alzheimer’s (AD), Parkinson’s (PD)], psychiatric [depression, anxiety, autism spectrum disorder (ASD)], functional gastrointestinal [irritable bowel syndrome (IBS), functional dyspepsia (FD)], cardiovascular (hypertension, atherosclerosis), and metabolic disorders (obesity, diabetes). This review critically analyses: (1) External factors driving dysbiosis; (2) Neural, immune, and metabolic communication pathways; (3) Molecular mechanisms of microbiota-brain crosstalk; (4) Disease-specific pathophysiological interactions; and (5) Therapeutic potential of microbiota modulation. Understanding the BGMA is pivotal for developing novel diagnostic and therapeutic strategies in gastroenterology and beyond.

## 2 Methodological approach

**Figure 1:**
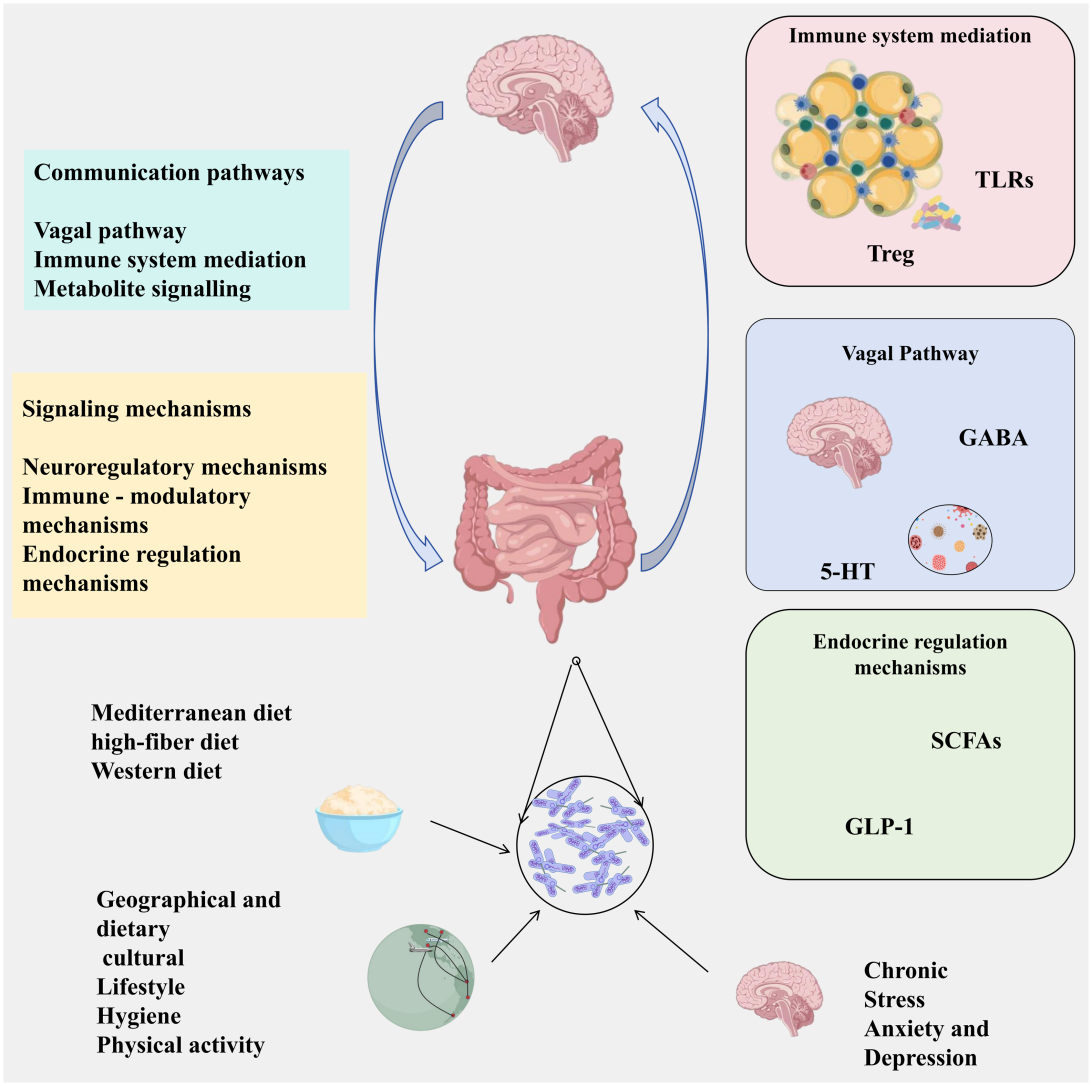
Core communication pathways and regulatory factors of the brain-gut-microbiota axis (BGMA). This figure integrates the bidirectional communication mechanism of BGMA, including: ① neural pathways (vagus nerve); ② immune-mediated pathways (cytokines, immune cell activation); ③ metabolite signaling pathways (SCFAs, tryptophan metabolites, bile acids, etc.). External factors (diet, psychological stress, environment, lifestyle) regulate these pathways by influencing the composition of the microbiota, thereby affecting the function of the central nervous system. Abbreviations: SCFAs, short-chain fatty acids; GABA, gamma-aminobutyric acid; 5-HT, 5-hydroxytryptamine; GLP-1, glucagon-like peptide-1; Treg, regulatory T cells. Created with BioGDP.com (Jiang et al., 2025).

**Figure 2:**
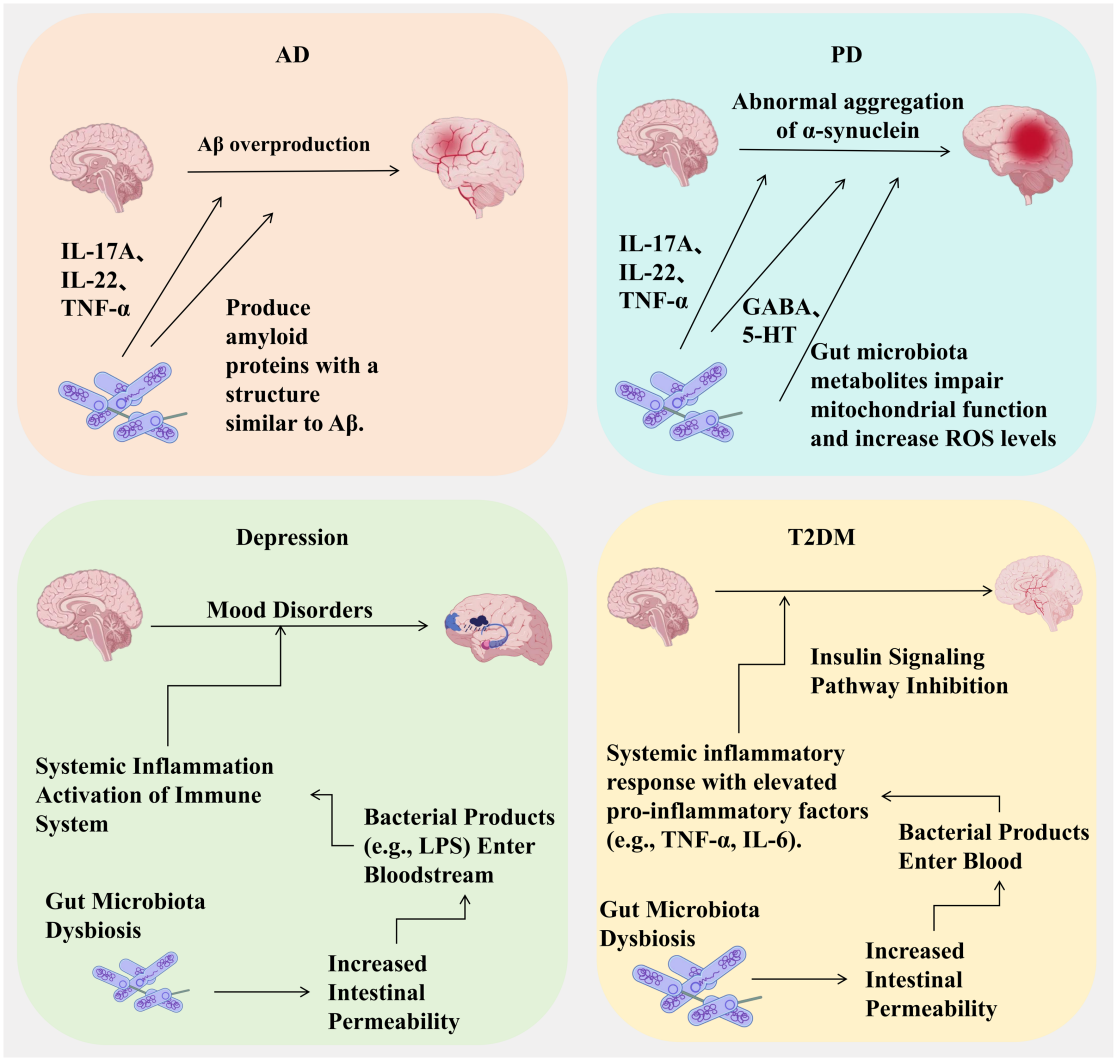
Core pathological mechanisms linking gut microbiota dysbiosis to four major diseases. This figure summarizes the mechanism of action of intestinal flora imbalance in Alzheimer’s disease (AD), Parkinson’s disease (PD), depression and type 2 diabetes mellitus (T2DM). AD: Flora imbalance promotes excessive production of Aβ and neuroinflammation (IL-17A, IL-22, TNF-α); PD: Flora metabolites (TMAO) induce abnormal aggregation of α-synuclein, mitochondrial dysfunction and oxidative stress; Depression: Intestinal leakage leads to LPS entering the blood, activating systemic immunity; T2DM: Flora imbalance triggers systemic inflammation and inhibits insulin signaling pathways. Abbreviations: Aβ, β-amyloid protein; TMAO, trimethylamine N-oxide; LPS, lipopolysaccharide. Created with BioGDP.com (Jiang et al., 2025).

**Figure 3:**
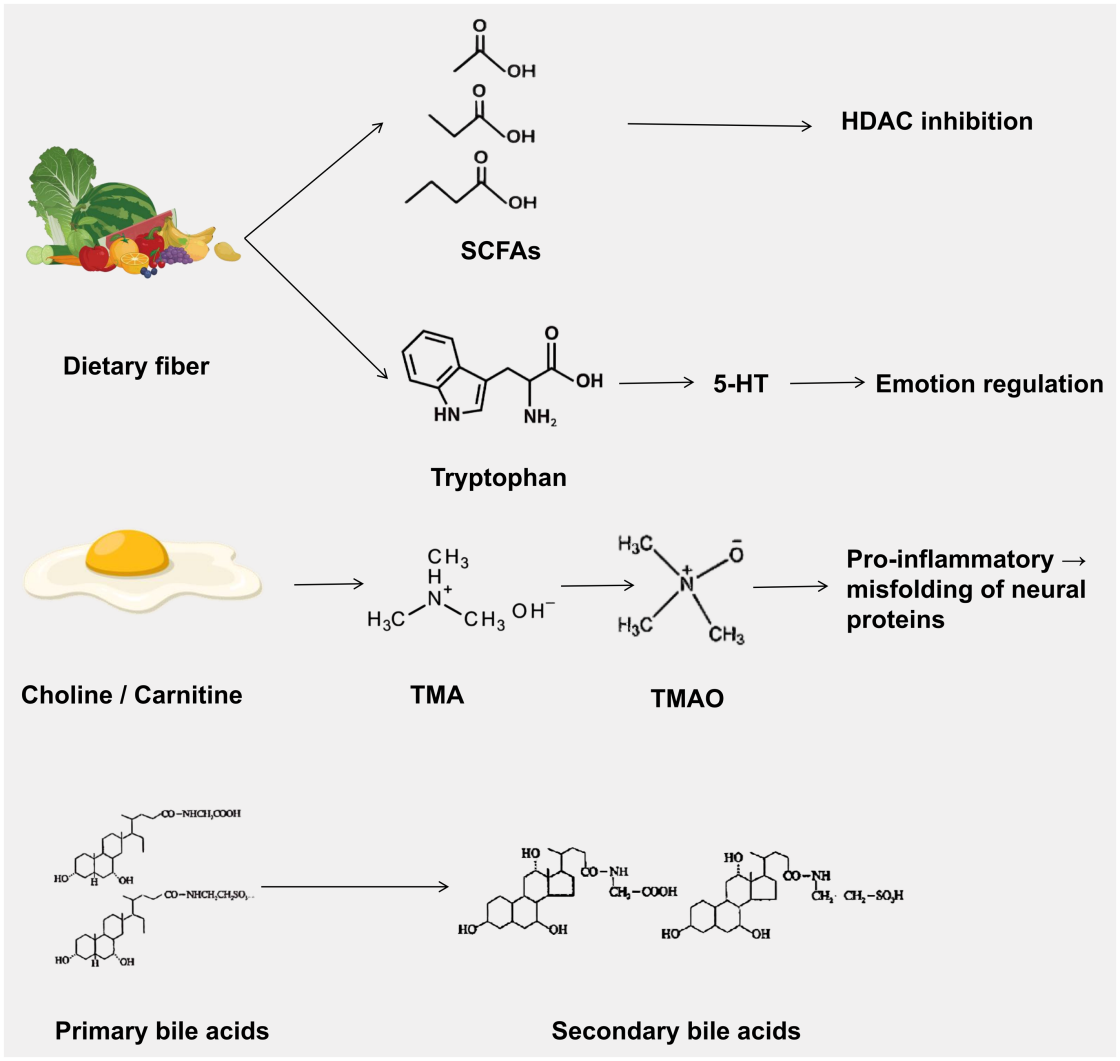
Major signaling pathways of microbiota-derived metabolites and their effects on host physiology. This figure illustrates four key microbial metabolites and their mechanisms of action: ① Short-chain fatty acids (SCFAs) produced by the fermentation of dietary fiber regulate immune function, blood-brain barrier integrity, and neuronal function through pathways such as HDAC inhibition and GPCR signaling; ② Tryptophan metabolism generates 5-hydroxytryptamine (5-HT), which participates in mood regulation; ③ Choline/carnitine is converted by microorganisms into trimethylamine (TMA), which is further transformed into trimethylamine N-oxide (TMAO), promoting neuroinflammation and protein misfolding; ④ Primary bile acids are converted into secondary bile acids, which regulate energy metabolism and neuroinflammation through FXR and TGR5 receptors. Abbreviations: HDAC, histone deacetylase; TMA, trimethylamine; TMAO, trimethylamine N-oxide; FXR, farnesoid X receptor; TGR5, G protein-coupled bile acid receptor 5.

This narrative review synthesizes peer-reviewed literature on the brain-gut-microbiota axis published between January 2000 and December 2023. We conducted systematic searches of PubMed/Medline, EMBASE, and Cochrane Library using keywords: (“brain-gut-microbiota axis” OR “microbiota-gut-brain axis”) AND (“dysbiosis” OR “neuroinflammation” OR “SCFAs” OR “probiotics” OR “fecal microbiota transplantation”). Inclusion criteria: (1) peer-reviewed original research and reviews; (2) English language; (3) human studies or relevant preclinical models. Quality assessment: RCTs were evaluated using Cochrane RoB 2; observational studies using Newcastle-Ottawa Scale. Data synthesis was narrative, organized by mechanistic pathways and disease categories (Figures 1–3).

## 3 Perturbation of gut microbiata by external factors

Before examining external factors that disrupt gut microbiota, it is essential to define a healthy state. A healthy gut microbiota forms a complex, diverse, and unique ecosystem vital to host health. Typically dominated by the phyla Bacteroidetes and Firmicutes, it also includes smaller proportions of Actinobacteria, Proteobacteria, and Verrucomicrobia, alongside methanogenic archaea (e.g., *Methanobrevibacter smithii*), eukaryotic organisms (primarily yeasts), and diverse phages (Rinninella et al., 2019; Van Hul et al., 2024).

This microbial composition evolves significantly across the lifespan. In neonates, the mode of delivery profoundly shapes the initial microbiota, which gradually transitions toward an adult profile during weaning. Throughout childhood, the microbiota stabilizes and incorporates butyrate-producing Firmicutes. By pre-puberty, it resembles the adult microbiota taxonomically and functionally, though often retains higher levels of bifidobacteria and certain anaerobes (Milani et al., 2017; Ferretti et al., 2018).

Multiple factors dynamically shape the gut microbiota, including delivery mode, diet, antibiotic use, genetics, and environmental exposures (Claesson et al., 2012). For instance, dietary changes can rapidly alter microbial populations, with high-fiber intake promoting beneficial bacteria proliferation in the short term. In contrast, the elderly often exhibit reduced microbiota diversity and stability, potentially linked to age-related immune decline (Claesson et al., 2011).

### 3.1 Effects of diet on gut microbiota

Diet profoundly shapes the gut microbiota, significantly altering its composition, function, and diversity.

Mediterranean Diets: The traditional Mediterranean diet increases beneficial bacteria (e.g., Prevotella, Roseburia) and boosts SCFA production (see section 4.3), decreases harmful metabolites, and enhances functional pathways linked to cardiovascular health (Jiménez-González et al., 2025; Perrone and D’Angelo, 2025).

High-Fiber and Plant-Based Diets: High-fiber intake elevates beneficial *Lactobacillus* and *Bifidobacterium* levels (Reynolds et al., 2020). Specific fiber components promote microbial fermentation, yielding SCFAs—key mediators of host physiology whose detailed mechanisms are discussed in section 4.3. (Akanyibah et al., 2025). Diets rich in plant polyphenols further encourage growth of beneficial bacteria (e.g., *Bifidobacterium*) and offer anti-inflammatory and cardioprotective benefits (Tkacz et al., 2025).

High-Protein Diets: Some dietary proteins reach the colon, where microbes metabolize them. This increases Bacteroides abundance and generates diverse metabolites, though some may be associated with disease pathways (Neis et al., 2015).

Ketogenic Diet: This high-fat, low-carb diet alters microbiota by reducing Actinobacteria and Firmicutes while increasing Bacteroides, also influencing immune responses. However, its long-term effects require further investigation (Rew et al., 2022).

Western Diet: Characterized by high animal protein and saturated fats, this pattern decreases microbial diversity and elevates disease risk (David et al., 2014).

### 3.2 Environmental impacts on gut microbiota

Environmental factors play a crucial role in shaping the gut microbiota, significantly influencing human health. Geographic and cultural dietary differences drive distinct global dietary patterns, leading to varied gut microbial profiles. Populations consuming Western diets—characterized by high levels of animal protein, saturated fats, and ultra-processed foods—typically exhibit reduced microbial diversity with Bacteroides-dominant communities (Rahman et al., 2022). Conversely, non-Western populations, especially those adhering to whole-plant-food-based diets, often possess higher microbial diversity and greater abundance of Prevotella (De Filippo et al., 2010). The Hadza, for example, harbor unique xylan-degrading microbiota reflecting their plant-based diet and specialized glycan metabolism (Schnorr et al., 2014).

Lifestyle shifts like travel and migration also impact gut ecology. While Irish travelers maintaining a Western diet retain microbial similarities to non-industrialized populations, immigrants adopting a Western diet in the US experience decreased microbial diversity, diminished fiber-degradation capacity, and elevated disease risk (Keohane et al., 2020; Vangay et al., 2018).

Furthermore, hygiene practices, antibiotic use, and physical activity levels are linked to gut microbiota changes (Thabet et al., 2024). These factors frequently interact, complicating research: antibiotics can profoundly alter microbial structure and function; hygiene differences affect microbial transmission and colonization; and physical activity may indirectly influence the gut microbiota through metabolic and immune pathways (d’Humières et al., 2024).

### 3.3 Impact of psychological factors on gut microbiota (other than SCFAs)

Beyond SCFAs—which are comprehensively addressed in section 4.3—gut microbiota produce a diverse array of signaling molecules that influence brain function.

A complex, bidirectional relationship exists between psychological states and the gut microbiota within human physiology (Jiang et al., 2018). Chronic stress, anxiety, and depression significantly disrupt the composition, diversity, and function of gut microbial communities (Ma et al., 2021).

Tryptophan metabolism represents another critical interface. Gut microbiota metabolize tryptophan through three principal pathways: (i) direct modulation of 5-HT synthesis in enterochromaffin cells (microbiota-modulated but host-executed, detailed in section 5.1); (ii) the kynurenine pathway producing neuroactive metabolites (kynurenic acid, quinolinic acid) that cross the BBB and participate in neurotransmitter regulation and neuroinflammation; and (iii) microbial tryptophanase generating indole derivatives that activate aryl hydrocarbon receptors (AhRs), exerting antioxidant and anti-inflammatory effects (Ye et al., 2021). Notably, the relative flux through these pathways—particularly the competition between 5-HT synthesis and kynurenine metabolism—has profound implications for CNS function.

Bile acid metabolism: Gut microbiota convert primary to secondary bile acids. These metabolites regulate energy metabolism, inflammatory responses, and BBB function via farnesoid X receptor (FXR) and TGR5 receptors.

Other microbial products: Microbiota-derived polyamines and *γ*-aminobutyric acid (GABA) may influence neuronal growth and function, though CNS penetration of these compounds remains debated.

## 4 Gut microbiota-brain communication pathways

### 4.1 Vagal pathway

The vagus nerve, the body’s longest cranial nerve, is critical for gut—brain communication. Its unique nerve fiber distribution comprises 80% afferent fibers, extending from the gut to the brainstem’s solitary nucleus, and 20% efferent fibers, creating a bidirectional circuit. In the gut, vagal afferent fibers are widely distributed in the mucosa, muscle layers, and around blood vessels, detecting environmental changes, including microbial metabolic activities and the physical and chemical properties of gut contents (Tables 1, 2).

**Table 1:** Evidence-based interventions for modulating the gut-brain axis.

Intervention	Representative agent(s)	Mechanism of action	Clinical evidence and outcomes	Manuscript reference
Probiotics	*Bifidobacterium longum* 1714 *Lactobacillus* spp.	- ↑SCFA/GABA production- ↑Tryptophan metabolism → serotonin synthesis- ↓Pro-inflammatory cytokines (IL-6, TNF-*α*)	- Depression: Improved cognitive performance and stress reduction in healthy volunteers;- Cognition: Improved cognitive performance;- Anxiety: Reduced stress-induced cortisol response	Allen et al., 2016 (Sec 7.4)
Prebiotics	Dietary fibers (Inulin) Oligosaccharides	- Microbial fermentation → ↑SCFAs (butyrate/propionate)- Butyrate: HDAC inhibition → neuroprotection- Propionate: GPR41/43 signaling → appetite regulation	SCFA-mediated benefits in IBS and improved metabolic parameters in T2DM	Reynolds et al., 2020 (Sec 3.1)
Fecal microbiota transplantation (FMT)	Healthy donor microbiota	- Ecological restoration- ↑SCFA-producing taxa (e.g., Faecalibacterium)- ↓TMAO-producing bacteria- Normalize F/B ratio	- AD models: 40-50% reduction observed in germ-free models - Depression: HAM-D (SMD = -1.21, 95% CI: -1.88 to -0.53)	Elangovan et al., 2022 (Sec 7.1)
Pharmacobiotics	Sodium oligomannate (GV-971)	- Suppresses phenylalanine and isoleucine neuroinflammation- Blocks IL-1β release via microglial inhibition	- Cognitive preservation in mild-to-oderate AD	Wang et al., 2019; Xiao et al., 2021 (Sec 7.1 & 7.4)

**Table 2:** Impact of external factors on gut microbiota composition and clinical consequences.

Factor category	Representative exposure	Microbiota alterations	Functional consequences	Associated diseases
Diet	Mediterranean diet	↑Prevotella, ↑SCFA producers	Anti-inflammatory effects, cardiovascular protection	Obesity, T2DM
	Western-style diet	↓Diversity, ↑Bacteroides dominance	Intestinal barrier impairment, ↑inflammation	IBS, Atherosclerosis
Psychological	Chronic stress	↓Lactobacillus/Bifidobacterium, ↑*E. coli*	↓Serotonin synthesis, HPA axis hyperactivity	Depression, Anxiety disorders
Environmental	Migration (Western diet adoption)	↓Diversity, ↓Fiber-degrading capacity	↑Metabolic dysregulation	Metabolic syndrome
Medical	Antibiotics	↓Faecalibacterium, ↑Antibiotic-resistant species	Immune tolerance disruption	*C. difficile* infection, Allergies

Gut microbiota and their metabolites activate vagal afferent terminals through various mechanisms. For instance, microbial-derived SCFAs and bile acids act on enterocytes, prompting the release of neurotransmitters including serotonin (5-HT)—whose microbial regulation is detailed in section 5.1—and prostaglandins, which subsequently activate vagal afferent fibers. When activated, electrical signals travel along these fibers to the brainstem and then to multiple brain regions, such as the amygdala, hippocampus, and prefrontal cortex, influencing mood and cognition (Jiang et al., 2024; Wang S. et al., 2019; Visekruna and Luu, 2021).

Extensive experimental studies support the importance of the vagal pathway. Animal studies show that vagotomised mice exhibit altered HPA axis responses to stress, increased anxiety-like behaviors, and disrupted gut microbiota (Sudo et al., 2004). Moreover, chemogenetic and optogenetic techniques have demonstrated that selectively modulating vagal afferent fibers can impact brain perception of gut microbial signals, thereby altering feeding behavior and emotional states (Chen Y. et al., 2023).

### 4.2 Immune system mediation

The gut, the body’s largest immune organ, plays a crucial role in the interaction between gut microbiota and the brain. Gut microbiota can trigger immune responses by activating immune cells within the gut, leading to the production of cytokines and other immune signaling molecules that enter the bloodstream and affect brain function. Under normal physiological conditions, gut microbiota and the immune system maintain a dynamic equilibrium. The immune system can recognize and tolerate commensal microbiota while effectively defending against pathogens. However, when dysbiosis occurs, such as overgrowth of harmful bacteria or reduction of beneficial ones, it can cause abnormal activation of gut immune cells. The gut contains diverse immune cells, including macrophages, dendritic cells, T lymphocytes, and B lymphocytes. These cells recognize molecular patterns of gut microbiota via pattern-recognition receptors (PRRs), such as lipopolysaccharides (LPS) and peptidoglycans, initiating immune responses (Takeuchi and Akira, 2010).

Once activated, these immune cells secrete various cytokines. These cytokines reach the CNS via three routes: (i) humoral transport across a compromised blood–brain barrier; (ii) activation of endothelial cells and perivascular macrophages; and (iii) neural signaling via vagal afferents. Central cytokine exposure activates microglia and astrocytes, propagating neuroinflammation with consequences for neuronal function, synaptic plasticity, and neurotransmitter balance. The intestinal barrier mechanisms governing LPS translocation are detailed in section 4.2. For example, in neurodegenerative diseases such as Alzheimer’s disease (AD) and Parkinson’s disease (PD) (Chen C. H. et al., 2023), gut microbiota dysbiosis and elevated peripheral and central inflammation levels are often detected, indicating that gut microbiota may contribute to disease development through immune—mediated neuroinflammatory pathways (Lobionda et al., 2019).

### 4.3 Metabolite signaling

Short-chain fatty acids (SCFAs)—primarily acetate, propionate, and butyrate—are the most extensively characterized microbiota-derived metabolites in BGMA communication. Produced via microbial fermentation of dietary fibers in the colon, SCFAs enter the circulation through monocarboxylate transporters (MCTs) and passive diffusion, exerting pleiotropic effects on host physiology (Schneider et al., 2024). SCFAs influence brain function through four principal mechanisms: (i) Blood–brain barrier (BBB) modulation: SCFAs regulate tight junction protein expression, preserving BBB integrity and restricting entry of peripheral inflammatory factors (Qu et al., 2024). (ii) G protein-coupled receptor (GPCR) signaling: Activation of GPR41 and GPR43 on enteroendocrine cells, immune cells, and neurons triggers downstream pathways that regulate neurotransmitter synthesis and release (Ye et al., 2021). (iii) Epigenetic regulation: Butyrate acts as a histone deacetylase (HDAC) inhibitor, influencing neuronal gene expression with implications for learning, memory, and neuroprotection (Duranti et al., 2020). (iv) Gut hormone stimulation: SCFAs induce release of glucagon-like peptide-1 (GLP-1) and peptide YY (PYY) from enteroendocrine cells (Strandwitz et al., 2019). These hormones act via endocrine and vagal pathways to modulate hypothalamic appetite circuits and brain activity, representing a key node whereby peripheral metabolic signals are transduced into central regulation of energy homeostasis.

Beyond these canonical actions, SCFAs promote regulatory T-cell differentiation via GPR43, contributing to intestinal immune homeostasis (Liu et al., 2023), and directly modulate microglial maturation and function, underscoring their role in CNS development and neuroinflammation (Loh et al., 2024). Butyrate, in particular, has been shown to inhibit Aβ fibrillization *in vitro* and to shift microglial phenotype toward an anti-inflammatory state, effects relevant to neurodegenerative diseases (see section 6.1).

Crucially, this signaling is bidirectional: central neuroendocrine outputs (e.g., via autonomic nerves and the HPA axis) reciprocally modulate gut physiology—altering intestinal transit time, mucus secretion, and nutrient availability—thereby reshaping the microbial metabolic environment and SCFA production capacity. This feedback loop is further elaborated in section 5.3 (Paiva et al., 2024).

Tryptophan metabolism represents another critical interface. Gut microbiota metabolize tryptophan through three principal pathways: (i) direct modulation of 5-HT synthesis in enterochromaffin cells (microbiota-modulated but host-executed, detailed in section 5.1); (ii) kynurenine pathway producing neuroactive metabolites (kynurenic acid, quinolinic acid) that cross the BBB and participate in neurotransmitter regulation and neuroinflammation; and (iii) microbial tryptophanase generating indole derivatives that activate aryl hydrocarbon receptors (AhRs),exerting antioxidant and anti-inflammatory effects (Ye et al., 2021). Notably, the relative flux through these pathways—particularly the competition between 5-HT synthesis and kynurenine metabolism—has profound implications for CNS function.

Beyond SCFAs and tryptophan metabolites, gut microbiota participate in bile acid metabolism, converting primary to secondary bile acids. These metabolites regulate energy metabolism, inflammatory responses, and BBB function via farnesoid X receptor (FXR) and TGR5 receptors. Additionally, microbiota-derived polyamines and *γ*-aminobutyric acid (GABA) may influence neuronal growth and function, though CNS penetration of these compounds remains debated.

## 5 From gut microbiota to signaling mechanisms in the brain

### 5.1 Neuroregulatory mechanisms

The gut microbiota significantly influences brain function through synthesis and modulation of key neurotransmitters and their precursors. 5-HT regulation represents a paradigmatic example of microbiota–host interaction. Although the host synthesizes 5-HT in enterochromaffin cells (ECs) via tryptophan hydroxylase 1 (Tph1), specific bacterial strains modulate this process by influencing substrate availability and EC function. For instance, Lactobacillus and Oscillospira upregulate tryptophan synthase genes, enhancing tryptophan availability for 5-HT synthesis (Liu et al., 2023; Loh et al., 2024). Conversely, some spore-forming bacteria promote EC 5-HT production through yet-undefined mechanisms. This microbial influence accounts for the observation that approximately 95% of the body’s 5-HT is produced in the gut, with gut microbiota playing a critical regulatory role (Mawe and Hoffman, 2013; Yano et al., 2015).

Beyond 5-HT, specific bacterial strains produce other neurotransmitters: Bifidobacterium and Lactobacillus generate GABA, the brain’s primary inhibitory neurotransmitter (Duranti et al., 2020; Strandwitz et al., 2019); certain Clostridium species produce dopamine precursors. Microbiota-derived GABA may reach the brain via circulation or neural pathways, potentially modulating anxiety and sleep, though direct CNS penetration in humans remains to be conclusively demonstrated. These microbial-derived neuroactive compounds may signal via local enteroendocrine cells, vagal afferents, or (controversially) direct CNS entry, though the latter remains mechanistically unresolved in humans.

Bidirectional nature of 5-HT signaling: The 5-HT axis is not unidirectional. Host-derived 5-HT, released into the gut lumen from ECs, provides luminal signaling that can influence microbial gene expression and community structure (Yano et al., 2015). This brain-to-gut 5-HT release is regulated by CNS activity and stress states, as discussed in sections 6.1 and 6.2, creating a feedback loop that integrates central and peripheral signals. Altered microbial 5-HT modulation is linked to mood disorders, with deficiencies associated with depression and anxiety (Loh et al., 2024). The intricate interplay between host and microbial 5-HT pathways underscores the complexity of BGMA communication and its relevance to psychiatric and gastrointestinal disorders.

### 5.2 Immune—modulatory mechanisms of gut microbiota

The gut microbiota is essential for the development and function of the intestinal immune system. It interacts with epithelial and immune cells to modulate host immunity. For instance, microbiota stimulate Toll-like receptors (TLRs) and other pattern-recognition receptors (PRRs) on innate immune cells, thereby maintaining mucosal barrier integrity and preventing pathogen invasion. They also drive mucosal immune development, including secretory IgA production. This critical role is evidenced in germ-free mice, which exhibit underdeveloped innate immunity and compromised gut barriers.

In adaptive immunity, microbiota influence T-cell differentiation and promote regulatory T-cell (Treg) development—crucial for immune tolerance (Yang et al., 2020). Microbiota further regulate B-cell function and antibody production, activating B cells in gut-associated lymphoid tissue (GALT) to secrete IgA for mucosal defense (Lee et al., 2020). Through cytokine network modulation and immune cell activation, microbiota maintain gut immune homeostasis.

This section focuses on microbiota-immune interactions within the gut mucosa that establish the permissive environment for systemic inflammation (section 4.2 describes CNS consequences). Dysbiosis disrupts intestinal homeostasis, increasing permeability (“leaky gut”) and enabling LPS translocation—the initiating event for peripheral inflammation. Microbiota-derived SCFAs, particularly butyrate, enhance regulatory T-cell differentiation via GPR43 and promote antimicrobial peptide production, maintaining barrier integrity. When compromised, LPS enters circulation, triggering the cytokine cascades and neuroinflammatory responses detailed in section 3.2. Clinically, Alzheimer’s disease patients show elevated pro-inflammatory gut bacteria, with direct correlations between gut dysbiosis severity and brain inflammation markers (Liu et al., 2020; Chakraborty and Laird, 2025).

### 5.3 Endocrine regulation mechanisms of gut microbiota

Building upon the SCFA mechanisms detailed in section 4.3, this section focuses on the neuroendocrine integration of these microbial signals. The SCFA-induced release of glucagon-like peptide-1 (GLP-1) and peptide YY (PYY) from enteroendocrine cells [described in section 4.3(iv)] represents a critical node whereby peripheral metabolic signals are transduced into central appetite and satiety regulation. These hormones act via endocrine pathways and vagal afferents to modulate hypothalamic feeding circuits, forming a key feedback loop between gut microbial metabolism and CNS control of energy homeostasis (Larraufie et al., 2018; van de Wouw et al., 2018).

Crucially, this signaling is bidirectional: central neuroendocrine outputs (e.g., via autonomic nerves and the HPA axis) reciprocally modulate gut physiology—altering intestinal transit time, epithelial barrier permeability, and nutrient availability—thereby reshaping the microbial metabolic environment and SCFA production capacity. This feedback loop integrates the mechanisms discussed in sections 4.3 and 5.1–5.2 (Dinan and Cryan, 2012; Łoniewski et al., 2021).

While the preceding sections have detailed how gut microbiota signals to the brain, emerging evidence indicates that this communication is bidirectional. The following section examines how the CNS, through neural and neuroendocrine pathways, reciprocally regulates the gut microbial ecosystem.

## 6 The brain’s reverse regulation of gut microbiota

The central nervous system (CNS) regulates gastrointestinal function and the enteric nervous system (ENS) predominantly via the autonomic nervous system (ANS) and the hypothalamic–pituitary–adrenal (HPA) axis. These neural and neuroendocrine pathways indirectly modulate the gut microbiota by altering the intestinal microenvironment—specifically, gastrointestinal motility, epithelial secretion, mucosal immune tone, and nutrient availability. This section reviews the key neural and neuroendocrine mechanisms through which the brain influences the composition and functional activity of the gut microbiota (Li et al., 2023; Brisinda et al., 2004).

### 6.1 Autonomic nervous system regulation of the intestinal microenvironment

The autonomic nervous system (ANS), comprising sympathetic and parasympathetic (primarily vagal) branches, exerts profound regulatory effects on gastrointestinal physiology—and thereby shapes the intestinal microbial ecology. These effects are mediated through three principal mechanisms: (i) modulation of gastrointestinal motility, (ii) regulation of mucus secretion, and (iii) modulation of intestinal immune responses.

Motility and the Migrating Motor Complex. The migrating motor complex (MMC), a fasting-state gastrointestinal motor pattern orchestrated by enteric ganglion neurons, is influenced by food intake frequency, sleep quality, and stress. Regular meals, adequate sleep, and low stress help maintain normal MMC cycling and gut homeostasis. Conversely, MMC disruption alters intestinal transit time, with significant consequences for microbial composition. Patients with slow-transit constipation exhibit reduced colonic giant migrating contractions, a key feature of constipation-predominant irritable bowel syndrome (IBS-C) (Soliman et al., 2024). In contrast, IBS-diarrhea (IBS-D) patients show increased giant migrating contractions and accelerated intestinal transit (Deloose et al., 2012). These transit abnormalities markedly alter the composition, distribution, and metabolic function of microbiota across different gut regions, disrupting microecological balance (Macfarlane and Dillon, 2007; Carabotti et al., 2015).

Mucus Barrier Regulation. The ANS plays a pivotal role in regulating the thickness and biochemical composition of the intestinal mucus layer. Secreted by colonic and small intestinal goblet cells, this dynamic barrier lubricates the epithelial surface, shields the underlying mucosa from luminal insults, and serves as a critical niche—providing nutrients and specific adhesion sites for commensal bacteria—thereby underpinning gut microbial homeostasis (Chiaranunt et al., 2023). By modulating epithelial secretory activity, the ANS exerts precise, bidirectional control over both the quantity and structural integrity of mucus, thereby shaping microbiota colonization patterns and community dynamics. Conversely, ANS dysfunction can impair mucus secretion—resulting in either hyposecretion or aberrant glycosylation—thereby compromising the physical and functional integrity of the mucus barrier, disrupting the ecological niche for resident microbes, and ultimately driving dysbiosis (Zhu et al., 2022).

Neuro-Immune Modulation. The ANS shapes the gut microbiota microenvironment by regulating epithelial immune activation. Directly, neurotransmitters and neuropeptides released from enteric neurons bind to specific receptors on gut-resident immune cells—including macrophages and mast cells—thereby modulating their functional activity (Caputi et al., 2017). For example, 5-HT engages HTR2A and HTR3A receptors on macrophages, enhancing prostaglandin E₂ (PGE₂) expression and thereby influencing local immune responses (De Vadder et al., 2018)Indirectly, the ANS regulates gastrointestinal motility and mucus secretion, which alters the physical proximity and duration of bacterial–immune cell interactions, ultimately affecting immune activation. Moreover, vasoactive intestinal peptide (VIP), secreted by enteric neurons, modulates mucosal secretion and epithelial barrier integrity, thereby reshaping the dynamic crosstalk between the gut microbiota and host immune cells (Talbot et al., 2020; Khalil et al., 2025; Berends et al., 2019).

### 6.2 Hypothalamic–pituitary–adrenal axis and stress-mediated dysbiosis

Beyond autonomic pathways, the HPA axis represents a major neuroendocrine route through which psychological states influence gut microbiota. Chronic psychological stress activates the HPA axis, elevating circulating cortisol levels. This hormone alters the gut microenvironment, compromises barrier integrity, and creates opportunities for pathogenic bacteria to proliferate while inhibiting beneficial strains. Studies in stressed animal models demonstrate significant decreases in beneficial *Bifidobacterium* and *Lactobacillus*, increases in potentially harmful bacteria such as *Escherichia coli*, and reduced overall microbial diversity (Herman et al., 2016). The mechanisms involve HPA axis-mediated changes in gut motility, mucus secretion, and immune function, which collectively alter microbial habitat conditions.

Host-derived 5-HT, released into the gut lumen from enterochromaffin cells, provides luminal signaling that may influence microbial gene expression and community structure—representing a reverse direction of the 5-HT signaling axis described in sections 2.3 and 5.1. This brain-to-gut 5-HT release is regulated by CNS activity and stress states, demonstrating bidirectional communication.

Catecholamines (noradrenaline and adrenaline), released during stress responses, provide direct chemical signals to gut microbiota. Some enteric pathogens can alter their proliferation in response to exogenous catecholamines; for example, noradrenaline stimulates growth of several enteric pathogen strains and enhances *Campylobacter jejuni* virulence (Sudo, 2019; Chen Y. et al., 2021; Cao et al., 2025). However, the effects of catecholamines on commensal organisms and the precise role of microbial signaling molecules in healthy and diseased states remain incompletely understood and require further investigation.

### 6.3 Integration: bidirectional feedback loops

Beyond the SCFA-mediated mechanisms detailed in sections 4.3, gut microbiota employ diverse molecular strategies for host communication. Quorum-sensing molecules—typically associated with inter-microbial coordination—can be recognized by host epithelial, immune, and neural cells, potentially modulating gut physiology without direct bacterial contact. Microbe-derived neuroactive compounds extend the repertoire beyond SCFAs. These include: (i) GABA and tryptophan metabolites (section 5.1); (ii) trace amines (tyramine, phenylethylamine) that may interact with trace amine-associated receptors (TAARs); and (iii) choline metabolites including trimethylamine (TMA), the precursor to trimethylamine N-oxide (TMAO). TMAO merits particular attention given its association with cardiovascular risk and potential neurotoxicity through protein misfolding promotion.

Cytokines and immunomodulatory molecules represent another signaling layer. Bacteria-derived extracellular vesicles, peptidoglycans, and lipoteichoic acids can activate pattern-recognition receptors on host cells, initiating immune responses described in section 3.2. These signals reach extragastrointestinal targets via: (i) neurosecretory pathways (vagal afferents, spinal routes); (ii) endocrine mechanisms (portal circulation, systemic distribution); and (iii) direct neural interaction within the enteric nervous system. Notably, secondary bile acids—microbially transformed from host-derived primary bile acids—activate TGR5 receptors to suppress microglial activation and modulate neuroinflammation, while also influencing neurotransmitter release and synaptic transmission. These mechanisms complement the SCFA actions on brain function described in section 3.3.

## 7 The gut-brain interaction in disease

The bidirectional communication pathways detailed in sections 3–5—spanning neural (vagal), immune, metabolic, and neuroendocrine routes—establish the BGMA as a critical homeostatic system. Dysregulation of these carefully orchestrated mechanisms, whether initiated by external factors (section 3) or central nervous system outputs (section 5), has been implicated in diverse pathological conditions. This section examines disease-specific manifestations of BGMA disruption, emphasizing the distinction between established associations and tentative causal inferences, particularly in human studies.

### 7.1 Neurodegenerative diseases

#### 7.1.1 Alzheimer’s disease

Gut microbiota alterations are consistently observed in AD, though causality remains to be fully established. Dysbiosis characterized by increased pro-inflammatory taxa (Escherichia/Shigella) and reduced anti-inflammatory species (*Faecalibacterium*) has been associated with disease severity (Liu et al., 2020; Chakraborty and Laird, 2025). Mechanistic studies in preclinical models suggest that such dysbiosis may activate the C/EBPβ-AEP pathway, potentially accelerating amyloidogenesis (Chen C. et al., 2021). Pro-inflammatory microbial metabolites may compromise blood–brain barrier integrity, enabling systemic inflammatory signals to access the CNS. Notably, germ-free APP/PS1 mice exhibit a 40–50% reduction in Aβ deposition relative to conventionally raised controls, indicating that the gut microbiota contribute to amyloid pathology in genetically susceptible hosts (Elangovan et al., 2022). Moreover, fecal microbiota transplantation (FMT) from healthy donors has been shown to ameliorate both amyloid pathology and cognitive deficits in murine models (Elangovan et al., 2022).

In humans, a Phase II clinical trial of sodium oligomannate (GV-971)—a marine-derived oligosaccharide that modulates gut microbiota composition and attenuates phenylalanine- and isoleucine-driven neuroinflammation—demonstrated modest cognitive improvement in patients with mild-to-moderate AD. However, these results await independent replication and validation in larger, well-controlled Phase III trials. While approved for clinical use in China, GV-971 remains under active investigation in other jurisdictions, with ongoing debate regarding its overall benefit–risk profile. Gut-derived butyrate exhibits multimodal neuroprotective effects in preclinical AD models: beyond the HDAC inhibition and anti-inflammatory actions described in section 4.3, butyrate directly inhibits Aβ fibrillization *in vitro* and modulates microglial phenotype toward an anti-inflammatory state. Whether these mechanistic benefits translate into clinically meaningful outcomes in human AD patients remains under active investigation.

#### 7.1.2 Parkinson’s disease

PD is characterized by a gut-centered pathophysiology that parallels its CNS manifestations. Notably, *α*-synuclein aggregation has been detected in the enteric nervous system (ENS) prior to CNS involvement in a subset of patients, suggesting a potential gut-to-brain propagation pathway—though this remains controversial and may represent independent parallel processes rather than sequential spread. Elevated circulating levels of trimethylamine N-oxide (TMAO)—a microbiota-derived metabolite generated from dietary choline and L-carnitine—have been associated with greater disease severity and may contribute to α-synuclein aggregation and neuronal toxicity (Hoyles et al., 2021). In certain cohorts, the correlation between plasma TMAO concentrations and clinical disease severity has been reported as *r* = 0.72 (Hoyles et al., 2021); however, this association warrants validation in prospective longitudinal studies with standardized TMAO measurement protocols. Alterations in gut microbiota composition are consistently observed in PD patients, including reduced abundance of SCFA-producing genera—such as *Faecalibacterium* and *Roseburia*—and increased representation of pro-inflammatory bacterial taxa. These dysbiotic shifts may compromise intestinal barrier integrity, thereby promoting systemic inflammation and potentially facilitating retrograde transport of pathological α-synuclein from the gut to the brain via the vagus nerve (Cryan and Dinan, 2012). Nevertheless, it remains unclear whether these microbial alterations represent primary drivers of PD pathogenesis or secondary consequences of disease-associated factors—including dietary modifications, pharmacological interventions (particularly levodopa, which alters gut motility and microbiota), and diminished physical activity. Longitudinal studies starting in prodromal phases (e.g., REM sleep behavior disorder) are needed to disentangle cause from consequence.

### 7.2 Psychiatric and functional gastrointestinal disorders

#### 7.2.1 Major depressive disorder

Depression is associated with characteristic gut microbiota alterations, including depleted *Faecalibacterium* and elevated Proteobacteria (Łoniewski et al., 2021; Valles-Colomer et al., 2019). These changes may impair GABA and 5-HT synthesis, though direct causal evidence in humans is limited. The mechanisms underlying this association likely involve multiple pathways detailed in preceding sections: compromised intestinal barrier permitting LPS translocation (section 4.2), HPA axis dysregulation (section 5.2), and altered tryptophan metabolism favoring kynurenine over 5-HT synthesis (section 3.3). Increased intestinal permeability (measured by elevated lactulose/mannitol ratios) has been reported in depressed individuals, potentially permitting systemic translocation of lipopolysaccharides and triggering microglial activation and neuroinflammation via TLR4 signaling (Dantzer et al., 2008; Everard et al., 2013; Ley et al., 2006). However, antidepressant medications themselves alter gut motility and microbiota composition, complicating causal inference. Importantly, these associations may be bidirectional: depression-related behaviors (poor diet, reduced physical activity, sleep disturbance) may alter microbiota composition, while microbiota changes may exacerbate depressive symptoms through the mechanisms described above. Current evidence does not permit definitive conclusions regarding causality or the directionality of these relationships (Łoniewski et al., 2021).

#### 7.2.2 Autism spectrum disorder

Children with ASD commonly present with gastrointestinal comorbidities and alterations in gut microbiota composition, including reduced abundance of *Bifidobacterium* spp. and increased abundance of *Clostridium* spp. However, it remains unclear whether these microbial changes represent primary pathogenic drivers or secondary consequences of factors such as restricted dietary intake, prior antibiotic exposure, or ASD-associated behavioral patterns (Romano et al., 2023). Definitive conclusions await well-designed longitudinal studies that rigorously control for potential confounders.

#### 7.2.3 Irritable bowel syndrome

IBS serves as a prototypical disorder of brain–gut–microbiota axis (BGMA) dysregulation, characterized by visceral hypersensitivity, aberrant gastrointestinal motility, and compositional and functional alterations in the gut microbiota. Distinct microbial signatures and shifts in microbial metabolite profiles have been reported in both constipation-predominant (IBS-C) and diarrhea-predominant (IBS-D) subtypes; however, substantial inter-individual heterogeneity and overlap between subtypes limit diagnostic specificity, and no microbiota-derived biomarker has yet been validated for routine clinical application (Karakan et al., 2021).

### 7.3 Metabolic and cardiovascular diseases

Type 2 Diabetes Mellitus and Obesity. Metabolic diseases are associated with microbiota-dependent alterations in energy harvest and metabolic signaling. Obesity has been linked to shifts in the Firmicutes/Bacteroidetes (F/B) ratio and increased efficiency of energy extraction from dietary substrates; however, this association remains inconsistent across human studies. Deficiency in short-chain fatty acids (SCFAs) may impair hypothalamic regulation of appetite via GPR41/GPR43 signaling pathways, thereby contributing to dysregulated energy homeostasis (Ley et al., 2006).

Type 2 diabetes is characterized by reduced abundance of the mucin-degrading bacterium *Akkermansia muciniphila*, which compromises glucagon-like peptide-1 (GLP-1) secretion and intestinal barrier integrity (Everard et al., 2013). Additionally, trimethylamine N-oxide (TMAO) has been implicated in diabetes-associated cardiovascular complications, including functional impairment of high-density lipoprotein (HDL) and promotion of atherosclerosis.

Hypertension. Individuals with hypertension exhibit decreased gut microbial diversity and altered SCFA production profiles. TMAO may contribute to vascular dysfunction; however, its independent contribution—distinct from other established cardiovascular risk factors—requires further clarification.

### 7.4 Therapeutic interventions: evidence and limitations

Microbiota-targeted interventions hold translational promise but warrant cautious interpretation of the current evidence base.

**Probiotics and Prebiotics.** Specific bacterial strains—including *Bifidobacterium longum* 1714—have demonstrated preliminary efficacy in improving cognitive performance and attenuating stress-related behaviors in early-phase human studies (Allen et al., 2016). Nevertheless, observed effects are highly strain-specific, dose-dependent, and typically modest in magnitude. Significant heterogeneity across studies—including variations in experimental design, outcome measures, and participant demographics—constrains the generalizability of findings and complicates cross-study comparisons.

Fecal Microbiota Transplantation (FMT). FMT effectively restores microbial diversity and is an established therapy for recurrent *Clostridioides difficile* infection. In psychiatric applications, meta-analyses of randomized controlled trials demonstrate that FMT significantly reduces depressive symptom severity (SMD = -1.21, 95% CI: -1.88 to -0.53), as measured by the Hamilton Depression Rating Scale (HAM-D) (Zhang et al., 2025). However, existing evidence is limited by small sample sizes, insufficient follow-up duration to assess effect durability, and a lack of consensus on optimal donor selection criteria, stool preparation protocols, and delivery routes. Moreover, long-term safety data remain incomplete, particularly regarding risks of pathogen transmission, immune modulation, and potential metabolic sequelae.

**Pharmaceutical Approaches**. Sodium oligomannate (GV-971) represents a novel therapeutic strategy designed to modulate the gut microbiota–brain axis in AD. Although phase II/III clinical trials yielded encouraging results, subsequent regulatory evaluations—including those by major international agencies—have emphasized the need for additional, rigorously designed confirmatory studies (Wang X. et al., 2019; Xiao et al., 2021). While approved for clinical use in China, GV-971 remains under active investigation in other jurisdictions, underscoring ongoing uncertainty regarding its overall clinical benefit–risk profile.

Critical Appraisal. Several methodological and biological limitations temper enthusiasm for microbiota-targeted therapies: (1) the majority of human trials are underpowered and of short duration (Ley et al., 2006); (2) placebo responses are robust and clinically meaningful in functional gastrointestinal and psychiatric disorders; (3) it remains unclear whether therapeutic benefits are best achieved through precise targeting of specific taxa versus broader modulation of community structure and function (Li et al., 2026); (4) substantial inter-individual variability in baseline microbiota composition and host–microbe interactions poses challenges for standardization and personalized application (Porcari et al., 2025).

## 8 Conclusion

The BGMA is a fundamental regulator of human physiology, with dysbiosis serving as a critical interface between environmental exposures and the pathogenesis of diverse diseases affecting the gut, brain, and systemic health. Key mechanisms involve disrupted neural signaling (vagus), immune activation (cytokine-mediated neuroinflammation), and altered microbial metabolite profiles (SCFAs, TMAO, tryptophan derivatives). Targeting the microbiota through dietary modification, probiotics, prebiotics, or FMT holds significant therapeutic promise, particularly for conditions like IBS, depression, and potentially neurodegenerative disorders where treatment options are limited. Translating BGMA research into clinical practice requires overcoming challenges in establishing causality, standardizing methodologies, and validating interventions in large human trials. Understanding and harnessing the BGMA represents a paradigm shift toward integrated, microbiota-informed approaches in gastroenterology, neurology, and psychiatry.
