# Correction: Brain-gut-microbiota axis: a review on the bidirectional regulatory mechanisms between gut microbiota and brain and their disease interactions

**DOI:** 10.3389/fmicb.2026.1927901

**Published:** 2026-07-22

**Authors:** Hui Shen, Si Yun Wang, Yan Yan Zhao, Jia Lin Zhou, Jie Zhao, Wei Kai Zhu

**Affiliations:** 1 Traditional Chinese Medicine Department, First Affiliated Hospital of Dalian Medical University, Dalian, Liaoning, China; 2 National-Local Joint Engineering Research Center for Drug Research and Development of Neurodegenerative Diseases, Dalian Medical University, Dalian, Liaoning, China; 3 College of Health-Preservation and Wellness, Dalian Medical University, Dalian, Liaoning, China; 4 Institute (College) of Integrative Medicine, Dalian Medical University, Dalian, Liaoning, China

**Keywords:** brain-gut-microbiota axis, gut microbiota, immune activation, microbial metabolites, neural pathways

Author Wei Kai Zhu was erroneously designated as a co-first author. Authors Hui Shen, Si Yun Wang and Yan Yan Zhao remain as co-first authors.

The order of author affiliations was incorrect upon publication. The correct affiliation order is as follows:

^1^Traditional Chinese Medicine Department, First Affiliated Hospital of Dalian Medical University, Dalian, Liaoning, China

^2^National-Local Joint Engineering Research Center for Drug Research and Development of Neurodegenerative Diseases, Dalian Medical University, Dalian, Liaoning, China

^3^College of Health-Preservation and Wellness, Dalian Medical University, Dalian, Liaoning, China

^4^Institute (College) of Integrative Medicine, Dalian Medical University, Dalian, Liaoning, China

The original version of this article has been updated.

